# Optically Enhanced Solid-State ^1^H NMR Spectroscopy

**DOI:** 10.1021/jacs.3c03937

**Published:** 2023-06-27

**Authors:** Federico De Biasi, Michael A. Hope, Claudia E. Avalos, Ganesan Karthikeyan, Gilles Casano, Aditya Mishra, Saumya Badoni, Gabriele Stevanato, Dominik J. Kubicki, Jonas Milani, Jean-Philippe Ansermet, Aaron J. Rossini, Moreno Lelli, Olivier Ouari, Lyndon Emsley

**Affiliations:** † Institut des Sciences et Ingenierie Chimiques, École Polytechnique Fedérale de Lausanne (EPFL), CH-1015 Lausanne, Switzerland; ‡ Institute of Radical Chemistry, Aix-Marseille University, CNRS, ICR, 13013 Marseille, France; § Institut de Physique, École Polytechnique Fedérale de Lausanne (EPFL), CH-1015 Lausanne, Switzerland; ∥ U.S. Department of Energy, Ames Laboratory, Ames, Iowa 50011, United States; ⊥ Department of Chemistry, Iowa State University, Ames, Iowa 50011, United States; # Magnetic Resonance Center (CERM) and Department of Chemistry “Ugo Schiff”, University of Florence, 50019 Sesto Fiorentino, Italy; ¶ Consorzio Interuniversitario Risonanze Magnetiche delle Metalloproteine Paramagnetiche (CIRMMP), 50019 Sesto Fiorentino, Italy

## Abstract

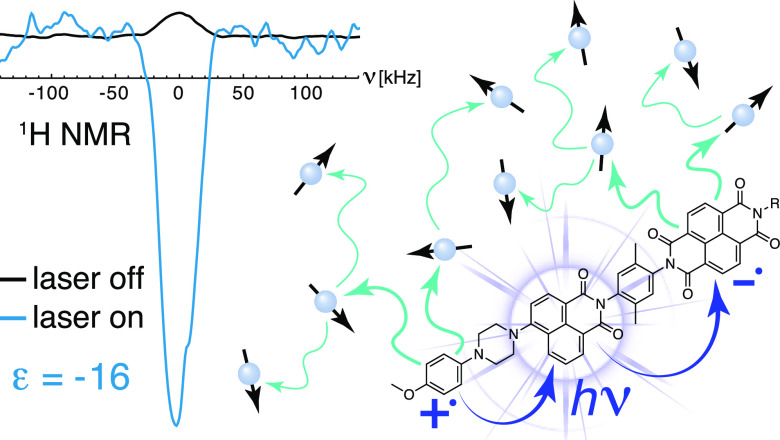

Low sensitivity is the primary limitation to extending nuclear magnetic resonance (NMR) techniques to more advanced chemical and structural studies. Photochemically induced dynamic nuclear polarization (photo-CIDNP) is an NMR hyperpolarization technique where light is used to excite a suitable donor–acceptor system, creating a spin-correlated radical pair whose evolution drives nuclear hyperpolarization. Systems that exhibit photo-CIDNP in solids are not common, and this effect has, up to now, only been observed for ^13^C and ^15^N nuclei. However, the low gyromagnetic ratio and natural abundance of these nuclei trap the local hyperpolarization in the vicinity of the chromophore and limit the utility for bulk hyperpolarization. Here, we report the first example of optically enhanced solid-state ^1^H NMR spectroscopy in the high-field regime. This is achieved via photo-CIDNP of a donor–chromophore–acceptor molecule in a frozen solution at 0.3 T and 85 K, where spontaneous spin diffusion among the abundant strongly coupled ^1^H nuclei relays polarization through the whole sample, yielding a 16-fold bulk ^1^H signal enhancement under continuous laser irradiation at 450 nm. These findings enable a new strategy for hyperpolarized NMR beyond the current limits of conventional microwave-driven DNP.

## Introduction

Nuclear magnetic resonance (NMR) spectroscopy is the method of choice to determine the atomic-level composition, structure, and dynamics in complex molecular or materials systems from pure solutions to disordered solids.^1−23^ This is due to the very rich chemical contrast provided by chemical shifts, which is largely orthogonal to other characterization methods. However, NMR is a low-energy technique, and its comparably low sensitivity is today the main roadblock to many applications, such as trace analysis, surface science, and metabolic imaging.

The sensitivity limitation of NMR is so critical in contemporary structural analysis that the development of methods to generate nuclear hyperpolarization is now of central importance.^24−27^ For solids, the most general approach so far is microwave-induced dynamic nuclear polarization (DNP).^26^ In this approach, a material under investigation is typically co-formulated with a stable radical and cooled to cryogenic temperatures. Then, upon microwave irradiation, the large thermal electron spin polarization is transferred from the radical spins to nearby nuclei and successively, by spin diffusion, to the entire nuclear spin network.^28−31^ In this framework, ^1^H nuclei are the ideal nuclear species to be polarized due to the very efficient spin diffusion arising from their large gyromagnetic ratio and high natural abundance.^31−35^

In recent years, optically driven nuclear polarization in solids has attracted more and more attention,^36−38^ primarily because of its potential to overcome the enhancement limits of standard microwave-induced DNP approaches imposed by the thermal electron polarization. Indeed, for ^1^H nuclei, the theoretical maximum DNP enhancement ε_max_, which corresponds to the ratio of the electron and proton gyromagnetic ratios, ε_max_ = |γ_e_/γ_1H_|, is a factor of 658.^26^ Optically induced nuclear polarization is not limited by this constraint, as photoexcited states can be generated with polarization much higher than equilibrium electron spin polarization^38−43^ that can, in theory, be transferred to nuclear spins.

Today, the most common optical nuclear polarization methods in solids harness the electron spin polarization in the excited triplet state of a chromophore, using gated microwave irradiation, fast magnetic field sweeps, and/or low-field level anti-crossing (LAC) matching conditions to transfer the electron spin polarization to nearby nuclear spins.^27,38,44−51^ These optical hyperpolarization techniques are typically performed in single crystals, as they require alignment of the chromophore molecular frame with respect to the applied magnetic field because of the strong orientation dependence of the triplet zero-field splitting. Consequently, it is extremely challenging to generate optical nuclear polarization if the target cannot be formulated as a single crystal, although one example of ^1^H hyperpolarization has been observed in pentacene-doped polycrystalline naphthalene under microwave irradiation and magnetic field sweeping.^52^

Photochemically induced DNP (photo-CIDNP) is an alternative technique that can be applied in the high-field regime without using microwaves.^53−55^ Despite the similarity in the name, spin polarization in photo-CIDNP is achieved through a fundamentally different process as compared to microwave-induced DNP. Originally, photo-CIDNP was observed for the products of photochemical reactions in solution^56,57^ and has since found application for the study of various radical photoreactions as well as the surface accessibility of proteins.^53,58,59^ However, solution-state photo-CIDNP is not appropriate as a general sensitivity enhancement method because there is no effective mechanism to transfer the local hyperpolarization to other species of interest.

In the solid-state, photo-CIDNP is caused by reaction cycles where a transient spin-correlated radical pair (SCRP) is created upon photoexcitation and whose evolution drives nuclear hyperpolarization.^54^ Local (site-specific) solid-state photo-CIDNP signal enhancements of up to 10,000 have been observed at 4.7 T in ^13^C NMR spectra of various biological systems,^60,61^ typically flavoproteins and photosynthetic reaction centers, and the effect has been studied under magic angle spinning at fields up to 17.6 T.^60,62^ Nevertheless, to date, only low-γ nuclei (^13^C and ^15^N) have been successfully directly polarized via this method in the solid state.^60,63−71^ In one example, ^13^C polarization was then transferred to adjacent protons by radiofrequency-driven cross polarization.^72^

As a result, solid-state photo-CIDNP has so far been used to hyperpolarize a target molecule and its immediate environment. Since ^13^C and ^15^N do not possess a large natural abundance, the magnetization generated by photo-CIDNP cannot propagate far by spin diffusion.^30,73,74^ In analogy to microwave-induced DNP methodologies, here, we propose the generation of ^1^H hyperpolarization by photo-CIDNP that can then be relayed throughout the bulk of the sample by spontaneous ^1^H–^1^H spin diffusion. This notably requires a suitably tailored photo-CIDNP polarizing agent, capable of generating net ^1^H hyperpolarization in the solid state.

**Figure 1:**
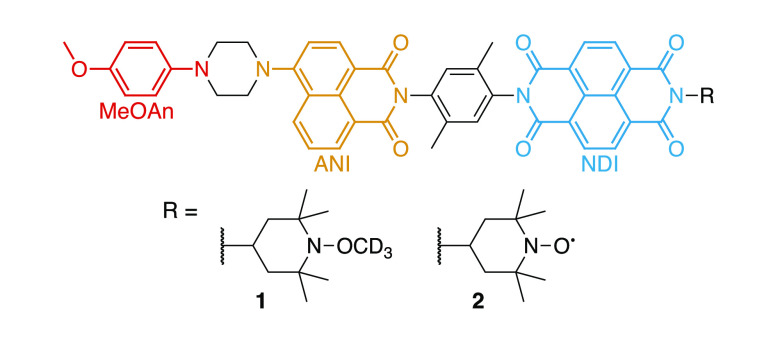
Structures of molecules used in this work. The donor (MeOAn), chromophore (ANI), and acceptor (NDI) moieties in the photoactive part of the structure are shown in red, yellow, and blue, respectively. The linker units are shown in black.

Here, we report the first example of direct ^1^H optical hyperpolarization in solids in the high-field regime. We achieved this through solid-state photo-CIDNP at 0.3 T (12.8 MHz) using a donor–chromophore–acceptor (D–C–A) molecule (Figure 1) as the polarizing agent. The method is microwave-free and leads to bulk ^1^H hyperpolarization. Notably, the effect does not rely on low-field LAC of the triplet levels, fast magnetic field sweeps, or aligned single crystals, and thus the method can in principle be extended to higher magnetic fields, best suited for high-resolution. Our findings represent a major step forward toward the general use of optical hyperpolarization in solid-state NMR spectroscopy.

To accomplish ^1^H solid-state photo-CIDNP, we chose a polarizing agent that is able to form a charge-separated SCRP upon photoexcitation and which generates a long-lived excited triplet state with coupled nuclear spins. We focused here on small molecules due to their larger versatility for future optimization compared to proteins and photosynthetic reaction centers.

The photoactive molecules (**1**) and (**2**) (Figure 1) satisfy the criteria above. They possess a D–C–A structure, where D is 4-methoxyaniline (MeOAn), C is 4-aminonaphthalene-1,8-dicarboximide (ANI), and A is naphthalene-1,8:4,5-bis(dicarboximide) (NDI). Here, we refer to this motif as PhotoPol. Two molecules were investigated, (**1**) and (**2**), which differ from each other by the presence of a stable nitroxide radical. Notably, molecule (**2**) has been extensively studied by Wasielewski et al.^39,75,76^ and was shown to generate electron spin polarization on the TEMPO radical upon photoexcitation. The photochemistry of similar donor–acceptor systems has also been studied both in the solid state and in solution,^41,77−80^ with some exhibiting ^1^H and ^13^C solution-state photo-CIDNP.^81,82^ Besides their donor–acceptor nature, molecules (**1**) and (**2**) were selected as ideal candidates to investigate ^1^H solid-state photo-CIDNP because of their unbalanced singlet and triplet SCRP charge recombination (CR) rates, as will be discussed further below.^75,79,80^

**Figure 2:**
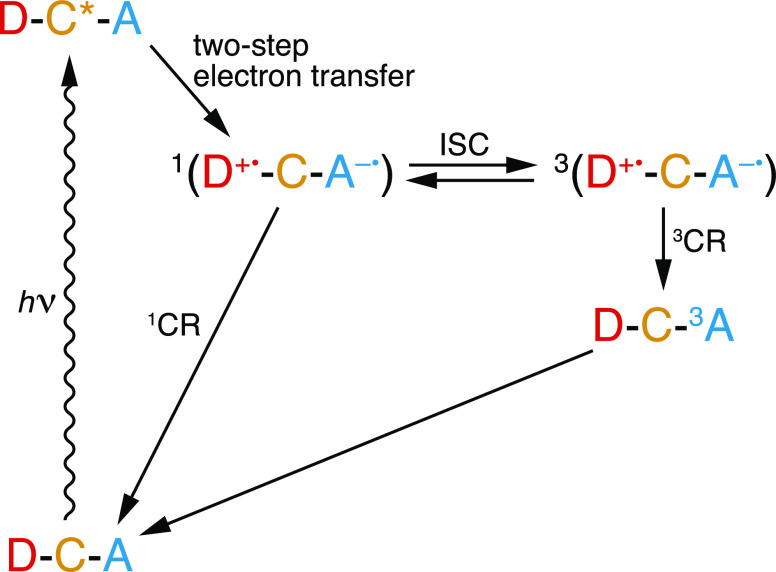
Photocycle of the D–C–A system in (**1**) and (**2**); *h*ν: photoexcitation, ISC: intersystem crossing, CR: charge recombination. The two-step electron transfer occurs on the picosecond timescale as D–C*–A → ^1^(D^+•^–C^–•^–A) → ^1^(D^+•^–C–A^–•^).

The photocycle kinetics of the D–C–A system are schematically depicted in Figure 2. After photoexcitation of the chromophore, charge separation in the form of a two-step intramolecular electron transfer generates a SCRP in the ^1^(D^+•^–C–A^–•^) singlet state,^39^ which undergoes radical-pair intersystem crossing (ISC) to also populate the ^3^(D^+•^–C–A^–•^) triplet state. Charge recombination can then occur in both the singlet (^1^CR) and triplet (^3^CR) channels, either returning to the ground state or populating the neutral triplet state, respectively. The neutral triplet state then also decays to the ground state at sufficiently long times. Interestingly, following the ^3^CR path, the triplet is localized on the acceptor (D–C–^3^A) and not the donor, as observed in toluene at 85 K for both (**2**) and one of its diamagnetic analogues (with R = 2,5-di-*t*-butylphenyl).^39,78^

In the following, we demonstrate that (**1**) and (**2**) generate enhanced ^1^H polarization upon continuous 450 nm illumination at 0.3 T. We then discuss the mechanisms for spin hyperpolarization.

## Results

A sample consisting of 10 μL of a 1 mM degassed solution of (**1**) in *o*-terphenyl (OTP) was prepared in a 3 mm outer-diameter NMR tube as described in Methods. OTP was chosen because of its relatively long ^1^H *T*_1_ relaxation time and its ability to form a clear and optically transparent glass, a necessary condition for optimal light penetration.^83−86^ The sample was melted at 65 °C and then rapidly frozen to 77 K in liquid N_2_ before insertion into the NMR probe to ensure good glass formation. ^1^H photo-CIDNP experiments were performed at 0.3 T and cryogenic temperatures (10–125 K) using a continuous wave (CW) 450 nm blue laser with adjustable output power. In the laser-on experiments, the laser is on for the whole duration of the experiments (Figure S1).

**Figure 3:**
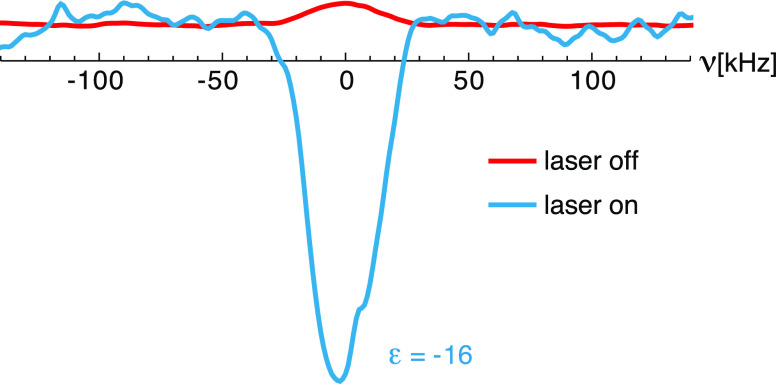
^1^H NMR spectra (12.8 MHz) of a 1 mM frozen solution of (**1**) in OTP at 85 K and 0.3 T without (red, 10^4^ scans) and with (blue, 50 scans) 3.8 W/cm^2^ CW laser illumination at 450 nm. A short (15 μs) solid echo pulse sequence was applied prior to signal acquisition and the re-polarization delay between scans was 20 s. More details about the NMR pulse sequence, acquisition parameters, and suppression of probe acoustic ringing^87^ are given in the Methods section and in the Supporting Information. (Note that the greater noise level in the laser-on spectrum is due to 200 times fewer scans being acquired).

Figure 3 shows the ^1^H NMR spectra of the sample recorded in the absence (red) and in the presence (blue) of 3.8 W/cm^2^ CW 450 nm laser irradiation. A clear inversion of the bulk ^1^H NMR signal is observed, with a signal enhancement factor of ε = −16 ± 2, defined as the ratio of the peak integrals with and without laser irradiation, ε = *I*_*z*,on_/*I*_*z*,off_. The inversion of the NMR signal on illumination can only be explained by a photo-CIDNP effect, as discussed in detail below.

**Figure 4:**
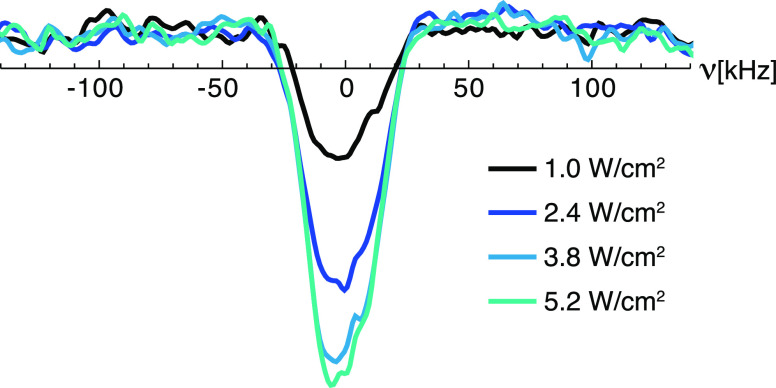
^1^H NMR spectra (12.8 MHz) of a 1 mM frozen solution of (**1**) in OTP (85 K and 0.3 T) recorded with CW laser illumination at 450 nm using different laser intensities (40 scans per experiment). The re-polarization delay between scans was 20 s.

Figure 4 shows the effect of varying the laser power on the photo-CIDNP enhancement. A relatively low intensity of 3.8 W/cm^2^ was sufficient to maximize the observed effect, which increases only minimally at higher powers. Even at the lowest power used, 1.0 W/cm^2^, inversion of the NMR signal is still achieved, with an enhancement of ε = −6 ± 1.

**Figure 5:**
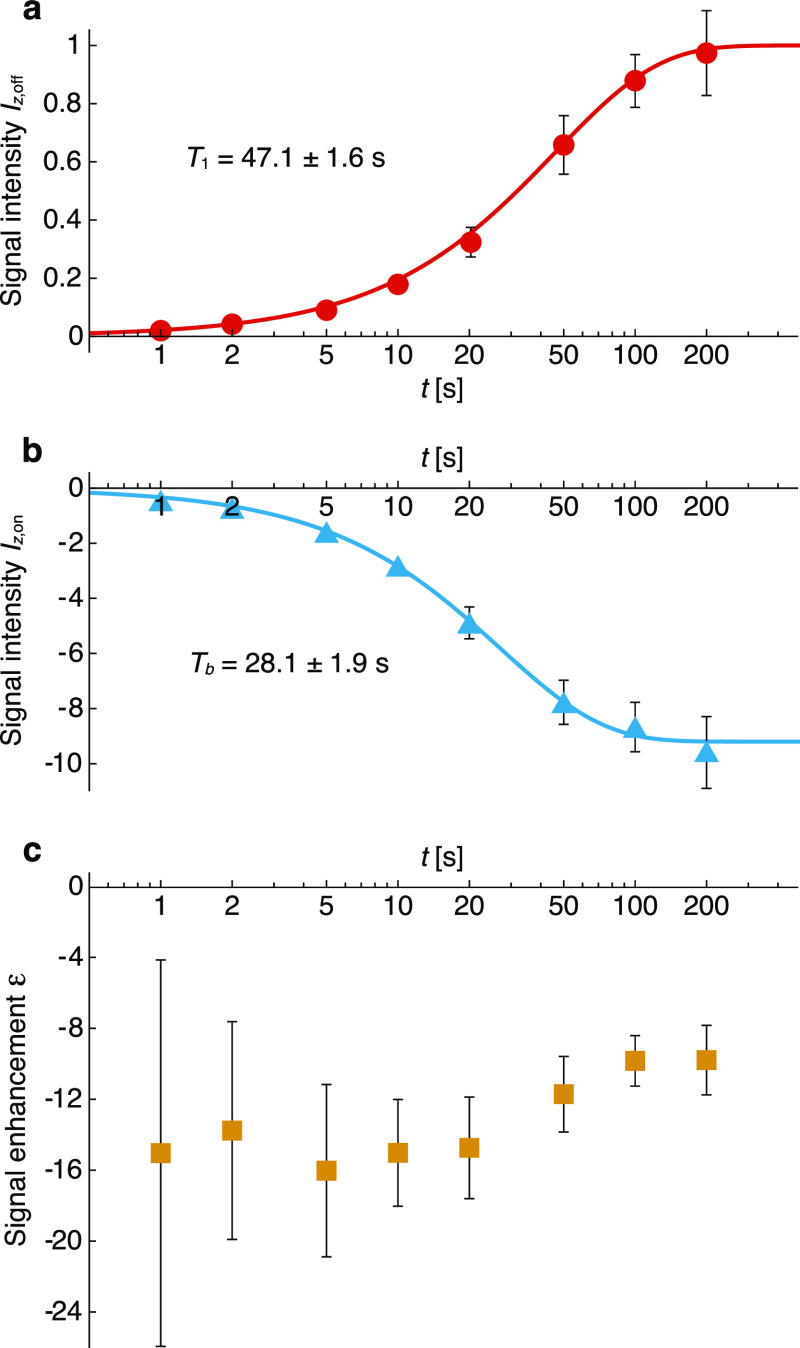
Saturation recovery experiments plotting the ^1^H NMR integrated signal intensity without light [*I*_*z*,off_, (a)], with 2.4 W/cm^2^ CW 450 nm light [*I*_*z*,on_, (b)], and the signal enhancement [ε = *I*_*z*,on_/*I*_*z*,off_, (c)], as a function of re-polarization delay at 85 K for a 1 mM frozen solution of (**1**) in OTP at 0.3 T. The number of scans for each datapoint is given in Table S1. Data were fitted (solid lines) with a single exponential function having a time constant *T*_1_ (longitudinal relaxation with no laser) or *T*_b_ (polarization buildup under CW irradiation) to yield the values given in the inset. All data shown here are background subtracted (see Supporting Information for additional details). Error bars are calculated from the signal-to-noise ratios in the spectra. Some of the errors in (a,b) are smaller than the symbol size.

^1^H longitudinal relaxation in the absence of light and polarization buildup under CW laser illumination were characterized on the same sample [1 mM of (**1**) in OTP] at 85 K (Figure 5). In both cases, the relaxation can be fit to an exponential function with time constants *T*_b_ and *T*_1_ with and without light, respectively. The buildup under illumination is significantly faster than without (*T*_1_ ≈ 1.7 *T*_b_). This effect is not due to sample heating induced by the laser because similar *T*_1_ and *T*_b_ time constants were measured upon heating the sample to 125 K (Figure S2). This indicates that the generation and propagation of ^1^H polarization by photo-CIDNP is faster than *T*_1_ relaxation, potentially enabling further time savings in these experiments.

From the measured *T*_1_ values, it is possible to estimate that the proton spin diffusion length, λ = , in OTP is approximately 190 nm (see Supporting Information),^30,88^ much larger than the average distance between molecules of (**1**) in a frozen solution at 1 mM concentration (7.3 nm, estimated using the Wigner–Seitz radius). This qualitative analysis indicates that the signal enhancement observed under laser irradiation is not limited by spin diffusion and that the matrix is polarized uniformly.^31^ The dependence of the signal enhancement on buildup time (Figure 5c) is characteristic of relayed hyperpolarization.^30,31,89^ The enhancement exhibits a broad maximum of ε = −16 ± 5 between 2 and 20 s and then reaches a steady-state value of ε = −9 ± 2 after about 100 s. This decrease in the magnitude of the enhancement at long buildup times confirms that the system is not spin diffusion limited.^31^

To further verify that the enhancement is limited neither by spin diffusion nor by the laser power, ^1^H photo-CIDNP experiments were repeated with a lower concentration of 0.1 mM (**1**) in OTP (Figure S4). Similar to the 1 mM sample, the estimated spin diffusion length is still larger than the average interparticle distance at 0.1 mM concentration (16 nm). The steady-state signal enhancement ε = −8 ± 1, measured with a re-polarization delay of 200 s, is virtually identical to that observed on the 1 mM sample (ε = −9 ± 2), confirming that uniform polarization of the matrix and homogeneous optical excitation of the chromophores are attained at both concentrations.

**Figure 6:**
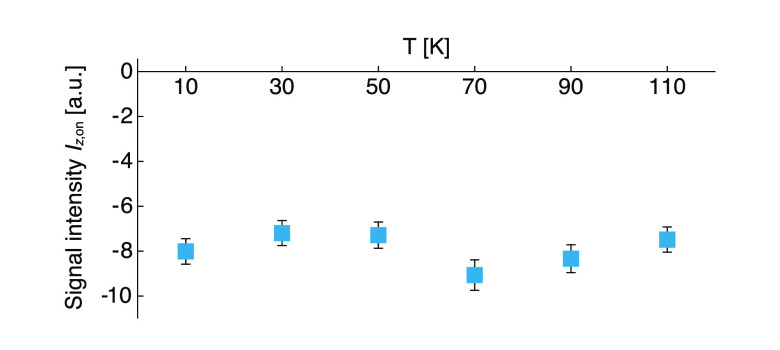
Steady-state ^1^H NMR integrated signal intensities in the presence of 3.8 W/cm^2^ CW 450 nm light as a function of temperature for a 1 mM frozen solution of (**1**) in OTP at 0.3 T. The re-polarization delay in the experiments performed at 90 and 110 K was 350 s; in all others it was 500 s (8 scans per experiment).

The effect of temperature on steady-state spin polarization generated by ^1^H photo-CIDNP was investigated in the 10–110 K range using a 1 mM frozen solution of (**1**) in OTP. Figure 6 shows the integrated signal intensities of the bulk ^1^H signal under CW laser illumination (*I*_*z*,on_) as a function of temperature. The negligible variation of *I*_*z*,on_ suggests that the net photo-CIDNP process is not affected by temperature within this range.

**Figure 7:**
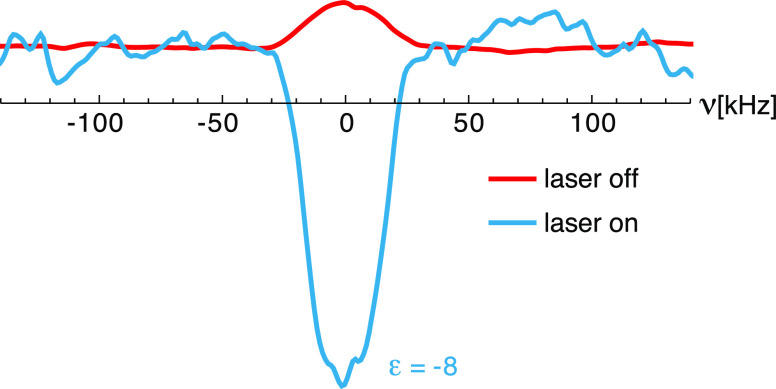
^1^H NMR spectra (12.8 MHz) of a 1 mM frozen solution of (**2**) in OTP at 85 K and 0.3 T without (red, 10^4^ scans) and with (blue, 60 scans) 3.8 W/cm^2^ CW laser illumination at 450 nm. The re-polarization delay between scans was 20 s.

^1^H NMR spectra in the presence and absence of light were also recorded on a 1 mM frozen solution of (**2**) in OTP. In this case, lower enhancements were observed than for (**1**). The maximum enhancement was ε = −8 ± 1 at 20 s (Figure 7), and the steady-state enhancement was ε = −5 ± 1, roughly half of that measured for (**1**) under similar conditions. Such differences are ascribed to the paramagnetic relaxation enhancement (PRE) and a spin diffusion barrier induced by the stable nitroxide radical in (**2**).^31,90−92^ Partially deuterating the matrix using a 20% OTP–80% OTP-*d*_14_ mixture did not improve the overall ^1^H photo-CIDNP enhancement (Figure S5), suggesting that the polarization transfer is also not spin diffusion limited for molecule (**2**). In contrast, with a toluene-*d*_3_ matrix a lower enhancement of ε = −0.7 ± 0.1 is observed, which is most likely due to faster ^1^H relaxation and/or poorer glass formation, though different solvents could in principle also fine tune the photochemistry of (**2**) (Figure S6).

## Discussion

The observation of the purely optically induced enhancements in Figures 3–7 unambiguously demonstrates that the PhotoPol motif can yield bulk ^1^H photo-CIDNP hyperpolarization in solids.

To enable future developments, it is then important to understand the mechanism behind this effect. Photo-CIDNP can occur by three mechanisms in the solid state: differential relaxation (DR), differential decay (DD), and three-spin mixing (TSM).^93−97^ We now briefly describe each mechanism and discuss their contribution in this system.

The simplest form of the Hamiltonian necessary to describe the photo-CIDNP effect in the SCRP-nuclear three-spin system is^98^
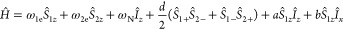
where ω_1e_ and ω_2e_ are the electron spin Larmor frequencies of the SCRP, ω_N_ is the nuclear spin Larmor frequency, *d* is the electron–electron interaction strength, and *a* and *b* are the secular and pseudo-secular components of the hyperfine interaction between the nucleus and the first electron spin. In general, this truncated form of the Hamiltonian describes the spin dynamics in the high-field regime (∼0.1 T and above), where the secular approximation is valid for all spin interactions except the hyperfine coupling for the nuclear spin.

The SCRP is typically singlet-born, but the singlet is not an eigenstate of the Hamiltonian, so the system coherently evolves between the |S⟩ =  (|αβ⟩ – |βα⟩) and |T_0_⟩ =  (|αβ⟩ + |βα⟩) electronic states,^98,99^ represented, respectively, by ^1^(D^+•^–C–A^–•^) and ^3^(D^+•^–C–A^–•^) in Figure 2. The |S⟩ ↔ |T_0_⟩ interconversion rate is given by the energy difference between the |αβ⟩ and |βα⟩ states, which, due to the hyperfine coupling, depends also on the spin state of the coupled nuclear spin. This results in nuclear spin sorting, where nuclear polarization is accumulated in |S⟩ and |T_0_⟩ with equal magnitude but opposite signs.^93,95^ If these states simply return to the ground state, the sorted nuclear spin states are recombined and no net nuclear hyperpolarization is observed.

In the DR mechanism, net nuclear polarization is generated by differences in nuclear relaxation between the singlet and triplet channels. Following charge recombination (^3^CR, Figure 2) to form the neutral triplet state (here, D–C–^3^A), PRE can reduce the nuclear polarization accumulated by spin sorting in the triplet channel if the polarized nucleus is close enough to the paramagnetic center. After the triplet D–C–^3^A then decays to the ground state, the nuclear polarization from the singlet and triplet channels no longer fully cancels, and net hyperpolarization from the singlet channel remains. In order to observe photo-CIDNP via DR, the neutral triplet lifetime must be at least comparable to the reduced nuclear *T*_1_, otherwise PRE is not effective. In addition, maximum polarization is expected when |Δω_e_| = |*a*/2| (where Δω_e_ = ω_1e_ – ω_2e_), as this is when the spin sorting mechanism is most efficient.^95^

In DD, nuclear hyperpolarization is observed after spin sorting if ^1^CR and ^3^CR (Figure 2) occur at different rates.^94^ When the nuclear Larmor frequency is comparable to half of the hyperfine coupling *a*, the pseudo-secular coupling *b* induces a LAC between the |αβα_N_⟩ and |αββ_N_⟩ states (or alternatively |βαα_N_⟩ and |βαβ_N_⟩, depending on the sign of *a*).^98^ This enables coherent nuclear spin flips (|α_N_⟩ ↔ |β_N_⟩) within both SCRP states, and such flips act to reduce the nuclear polarization accumulated in those states by spin sorting. The rate of spin flipping is equal and opposite in the singlet and triplet SCRP states; however, if CR happens more quickly in one of the channels, then nuclear polarization in that channel decreases less than in the other before the state decays and net hyperpolarization develops. Photo-CIDNP due to DD is observed if |Δω_e_|, |ω_N_|, and |*a*/2| are all comparable within 2 orders of magnitude,^94^ and maximum polarization is expected when |ω_N_| = |*a*/2|, as this maximizes the mixing of the states induced by the LAC.

In the TSM mechanism, nuclear hyperpolarization is generated by coherent evolution of the SCRP between the |S⟩ and |T_0_⟩ states accompanied with a concurrent nuclear spin flip (e.g., |Sα_N_⟩ ↔ |T_0_β_N_⟩), so that unbalanced polarizations are directly accumulated in ^1^(D^+•^–C–A^–•^) and ^3^(D^+•^–C–A^–•^), unlike the pure spin sorting discussed above.^96,97,100^ In the limit of strong coupling (|*d*| ≫ |Δω_e_|), this occurs when |ω_N_| = .^98,101^ Alternatively, in the weak coupling limit (|*d*| ≪ |Δω_e_|), TSM requires a double matching condition |Δω_e_| = |ω_N_| = |*a*/2|.^96,97,101^

The sign of the photo-CIDNP enhancement for each mechanism is determined by simple rules, depending on the signs of the secular component of the hyperfine coupling (*a*), the electron–electron interaction (*d*), and the difference of the electron Larmor frequencies (Δω_e_).^98^ The magnetic interactions in the PhotoPol motif have been previously determined through transient absorption experiments, (time-resolved) electron paramagnetic resonance spectroscopy, and density functional theory calculations.^39,75,77−80^ With these known values, we can consider the contribution of each mechanism.

The isotropic *g*-factor is higher for D^+•^ than for A^–•^;^39,78^ consequently, with a singlet-born SCRP and with PRE on A in the triplet channel, a negative hyperfine coupling is required for ^1^H nuclei on A^–•^ to give an overall negative enhancement by DR. This is indeed the case for both aromatic protons in NDI,^82^ and the magnitude of the hyperfine couplings (∼5.5 MHz) is comparable to 2|Δω_e_| ≈ 2.5 MHz at X-band. Furthermore, the lifetime of D–C–^3^A is in principle long enough for PRE to occur (∼42 μs, as measured in a toluene solution at room temperature),^77^ therefore we conclude that DR is a feasible mechanism here. However, if the predominant polarization mechanism was DR, the negligible temperature dependence of the photo-CIDNP effect shown in Figure 6 would require a constant triplet lifetime within the 10–110 K temperature range, which is unlikely.

From solution-state experiments,^79,80^ the measured ^3^CR for (**1**) is faster than ^1^CR, so DD is in principle a possible photo-CIDNP process here. Based on the isotropic *g*-factors for D^+•^ and A^–•^, negative enhancement would be obtained for protons on D^+•^ with negative hyperfine couplings and/or protons on A^–•^ with positive hyperfine coupling, when their magnitude is comparable to 2|ω_N_| ≈ 25 MHz (^1^H at 0.3 T). However, as mentioned in the previous paragraph, A^–•^ has only negative hyperfine couplings, while D^+•^ has mainly positive calculated hyperfine couplings in the 5–40 MHz range,^39^ so we conclude that DD is unlikely to be the dominant mechanism and could in fact reduce the net negative enhancement.

In the case of TSM, a negative ^1^H enhancement is predicted for the measured negative electron–electron coupling in the PhotoPol moiety, *d* ≈ −5.5 MHz .^39,78,98^ Both *d* and Δω_e_ are of comparable magnitudes and depend on orientation, rendering this semi-quantitative analysis challenging. Nevertheless, the system most closely resembles the strong-coupling regime, for which the matching condition |ω_N_| =  can be satisfied with hyperfine coupling constants of ∼25 MHz, which is within the range of calculated values for D^+•^.^39^ The coherent dynamics of TSM depend only on the strength of the spin interactions, not on time-dependent decay and relaxation processes (c.f. DD and DR), assuming that the SCRP lifetime is long enough. The temperature dependence of the spin interactions for a molecule in a rigid frozen matrix is expected to be minimal in the range 10–100 K, consistent with the negligible temperature dependence below 110 K in Figure 6.

Overall, based on the sign analysis, matching conditions, and temperature dependence, we propose that TSM is most likely to be the dominant photo-CIDNP mechanism here, although both DR and DD could also contribute.

Finally, here we have demonstrated the proof of principle for bulk ^1^H hyperpolarization by photo-CIDNP in the solid state. However, we note that the magnitude of the effect using the PhotoPol motif is relatively small compared to previously reported ^13^C and ^15^N solid-state photo-CIDNP enhancements at higher fields in proteins.^60,63−71^ We speculate that this could be due to cancellation between competing pathways with opposite signs, as well as dependence of the magnetic interactions on orientation, especially since ^1^H spin diffusion acts to homogenize the overall polarization of the sample.

Having established bulk solid-state ^1^H hyperpolarization in the high-field regime, the next step is to extend the effect to the higher magnetic fields required for high-resolution NMR. The PhotoPol motif was chosen because of its well-characterized photochemistry, enabling the photo-CIDNP effect to be predicted and rationalized. However, based on their spin chemistry, molecules (**1**) and (**2**) are not expected to work at higher fields, for which alternative polarizing agents must be developed. Nevertheless, this can be achieved by tailoring the spin interactions to the desired field, and there is no intrinsic reason for photo-CIDNP to be less effective at high field (unlike microwave-driven DNP). Indeed, solid-state photo-CIDNP has been demonstrated for other nuclei across the whole range of NMR fields, from 0.3 to 17.6 T. We envisage that this optical hyperpolarization method can be readily extended to high-resolution NMR.

## Conclusions

Here, we have reported the first example of optically enhanced solid-state ^1^H NMR spectroscopy in the high-field regime. This was achieved via photo-CIDNP at 0.3 T using PhotoPol, a donor–chromophore–acceptor system, as the polarizing agent. The ^1^H hyperpolarization is relayed from the randomly oriented polarizing agent by spin diffusion to the bulk solvent (here OTP), resulting in uniform polarization across the entire sample. Therefore, unlike for nuclei with a low natural abundance, the polarization can be used to enhance a separate target molecule or solid, and/or transferred to heteronuclei of interest via cross-polarization. This opens up a new pathway toward hyperpolarized solid-state NMR spectroscopy beyond the thermal limit of microwave-driven DNP.

Given that localized photo-CIDNP enhancements have been reported of up to 10,000 for other nuclei,^60,61^ there is clearly tremendous room for further development of the relatively modest bulk ^1^H signal enhancements observed here. In particular, we note that based on our mechanistic analysis above, the versatile D–C–A polarization agent can almost certainly be tailored to optimize the hyperfine couplings, electron *g*-factors, and electron–electron couplings to increase the ^1^H polarization further and to extend the effect to higher magnetic fields. This will be achieved through a deeper understanding of the spin dynamics among the excited states, ultimately guiding a rational design of the molecular machinery for optimal spin chemistry.

## Methods

### Synthesis of Molecules (**1**) and (**2**)

See the Supporting Information for full details.

### Sample Preparation

Separate 1 mM solutions of (**1**) and (**2**) were prepared in toluene and stored at −80 °C. For (**1**), a solution at 0.1 mM concentration was also prepared. For each sample, 10 μL of the desired solution was transferred to the bottom of a 3 mm outer-diameter NMR glass tube and evacuated until all toluene evaporated (toluene was chosen because of its solvation properties and low boiling point). Then, 11 mg of solid OTP (*d* = 1.1 g/cm^3^) was added to the tube and melted at 65 °C. Several freeze–pump–thaw cycles using liquid N_2_ were applied to degas the sample, melting the OTP solution each time under vacuum with a water bath at 65 °C. Degassing was performed until no bubbles were observed during remelting of the OTP solution. After this, the samples were sealed with a flame torch and stored at −80 °C.

### NMR Spectrometer

The applied magnetic field of 0.3 T is generated using a Varian electromagnet. The NMR signal is recorded with a setup consisting of a PulseBlaster Spincore pulse generator, a PTS 620 frequency synthesizer, a TOMCO RF pulse amplifier, a Gage Applied RazorMax digitizer, and a home-built spectrometer. The setup is controlled with a LabVIEW interface. The NMR coil has a saddle geometry and is shielded by a copper cavity. The cavity is located inside a Bruker ER 4118CF cryostat connected to either a liquid N_2_ or a liquid He dewar with active temperature regulation. The tuning and matching capacitors (NMTIM120CEK, Municom) are situated outside of the cryostat in an aluminum box. Both the copper cavity and the cryostat have an optical window for sample irradiation, performed with a 450 nm CW blue laser coupled to a λ/2 waveplate and a polarizing beam splitter to adjust the output power. Light is delivered on the optical window of the cryostat using an optical fiber connected to a collimator. The diameter of the beam exiting the collimator is 3 mm, which corresponds to a cross-sectional area of 7 mm^2^. With our setup, we expect light losses due to the various interfaces that the beam crosses between the collimator and the sample, so the actual laser intensity at the sample position is likely to be much smaller than the values reported in the text and that are measured at the exit of the optical fiber.

### NMR Experiments

The pulse sequence used to record all NMR spectra is reported in Figure S1. The ^1^H resonance in Figures 3, 4, and 7 is centered at 12.765 MHz. Suppression of probe acoustic ringing was achieved by applying an additional inversion pulse only on even scans prior to the solid echo and inverting the receiver phase. The 90 and 180° pulses were 3 and 6 μs long, respectively. All spectra in Figures 3, 4 and 7 were acquired with τ_sat_ = 0.1 ms, τ_rec_ = 20 s, τ_rs_ = 0.5 ms, and τ_SE_ = 15 μs. Data in Figures 5 and 6 were acquired with identical parameters but varying τ_rec_ between 0.5 and 200 s (Figure 5) or with τ_rec_ either equal to 350 or 500 s (Figure 6). Presaturation was performed with a sequence of 20 equally spaced hard 90° pulses.

## Supporting Information Available

Pulse sequence and probe acoustic ringing suppression; additional buildup experiments and data fitting; calculation of the spin diffusion length in OTP; steady-state photo-CIDNP experiments on (**1**) at 0.1 mM in OTP; NMR experiments in other solid matrices; and synthesis of compounds (**1**) and (**2**) (PDF)
