# Supplementary material for: Optically Enhanced Solid-State 1H NMR Spectroscopy
Source: J Am Chem Soc. 2023 Jun 27;145(27):14874–83. doi: 10.1021/jacs.3c03937 (PMC10347552; doi:10.1021/jacs.3c03937)
Supplement: Supplementary file 1 — ja3c03937_si_001.pdf [file ja3c03937_si_001.pdf]

Optically enhanced solid-state ^{1}H NMR spectroscopy

Federico De Biasi,[1] Michael A. Hope,[1] Claudia E. Avalos,[1,†] Ganesan Karthikeyan,[2] Gilles Casano,[2] Aditya Mishra,[1] Saumya Badoni,[1] Gabriele Stevanato,[1,+] Dominik J. Kubicki,[1,‡] Jonas Milani,[3] Jean-Philippe Ansermet,[3] Aaron J. Rossini,[4,5] Moreno Lelli,[6,7] Olivier Ouari,[2] and Lyndon Emsley[1,*]

[1] *Institut des Sciences et Ingenierie Chimiques, École Polytechnique Fedérale de Lausanne (EPFL), CH-1015 Lausanne, Switzerland*

[2] *Institute of Radical Chemistry, Aix-Marseille University, CNRS, ICR, 13013 Marseille, France*

[3] *Institut de Physique, École Polytechnique Fedérale de Lausanne (EPFL), CH-1015 Lausanne, Switzerland*

[4] *U.S. Department of Energy, Ames Laboratory, Ames, Iowa 50011, United States*

[5] *Department of Chemistry, Iowa State University, Ames, Iowa 50011, United States*

[6] *Magnetic Resonance Center (CERM) and Department of Chemistry "Ugo Schiff", University of Florence, 50019 Sesto Fiorentino, Italy*

[7] *Consorzio Interuniversitario Risonanze Magnetiche delle Metalloproteine Paramagnetiche (CIRMMP), 50019 Sesto Fiorentino, Italy*

Raw NMR Data

All the raw NMR data associated with the manuscript can be accessed at the following link DOI: www.doi.org/10.5281/zenodo.8033136 and is available under the CC-BY-4.0 (Creative Commons Attribution-ShareAlike 4.0 International) license.

Summary

1) Pulse sequence and probe acoustic ringing suppression....S2
2) Additional buildup experiments and data fitting....S2
3) Calculation of the spin diffusion length in OTP....S4
4) Steady-state photo-CIDNP experiments on (**1**) at 0.1 mM in OTP....S4
5) NMR experiments in other solid matrices....S4
6) Synthetic procedures....S5
    6a) Synthesis of compound **1**....S5
    6b) Synthesis of compound **2**....S10

1) Pulse sequence and probe acoustic ringing suppression

All the NMR experiments reported here have been acquired using the pulse sequence in Fig. S1. Suppression of probe acoustic ringing was achieved by applying an additional inversion pulse (indicated as an open rectangle in Fig. S1) prior to the solid echo on even scans only, and inverting the receiver phase. In this way, only the signal is added during the phase cycling while the severe baseline distortions caused by acoustic ringing are removed. ^{1}H solid-state photo-CIDNP experiments have been performed by irradiating the sample with a continuous wave (CW) 450 nm blue laser with adjustable output power for the entire duration of the pulse sequence. Presaturation of the NMR signal before the re-polarization delay (τ_{rec}) was achieved with a series of equally spaced hard 90° pulses.

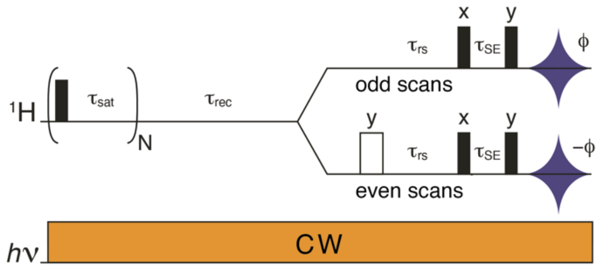

Figure S1. Pulse sequence used for signal acquisition and probe acoustic ringing suppression. Full and open rectangles represent hard 90° and 180° pulses, respectively. The inversion pulse is applied on even scans only. A two-step phase cycling is implemented for the receiver phase. All spectra in the main text were acquired with N = 20, τ_{sat} = 0.1 ms, τ_{rs} = 0.5 ms and τ_{SE} = 15 µs. τ_{rs} was chosen long enough so the acoustic ringing response during signal acquisition is not affected by the 180° pulse applied on even scans.

2) Additional buildup experiments and data fitting

After the buildup experiments at 85 K, additional buildup curves for 1 mM (**1**) in OTP with and without 2.4 W/cm^2 laser irradiation were measured at 125 K (Fig. S2) as mentioned in the main text. Note that some sample degradation occurred while measuring the laser-on buildup at 125 K for 5 hours, resulting in smaller enhancement factors (Fig. S2b) than those measured at 85 K for similar re-polarization delays (Fig. 5b). Degradation was further confirmed by cooling the sample back to 85 K and observing ^{1}H solid-state photo-CIDNP enhancements equal to about a half of those measured under identical conditions before acquiring the experiments at 125 K. On the contrary, sample degradation upon laser irradiation at 85 K was barely noticeable.

To minimize the bias induced by sample degradation in estimating T_b at both 85 K and 125 K, the laser-on buildup curves were measured by shuffling the re-polarization delays. The order of the delays is reported in Table S1, together with the number of scans for each experiment.

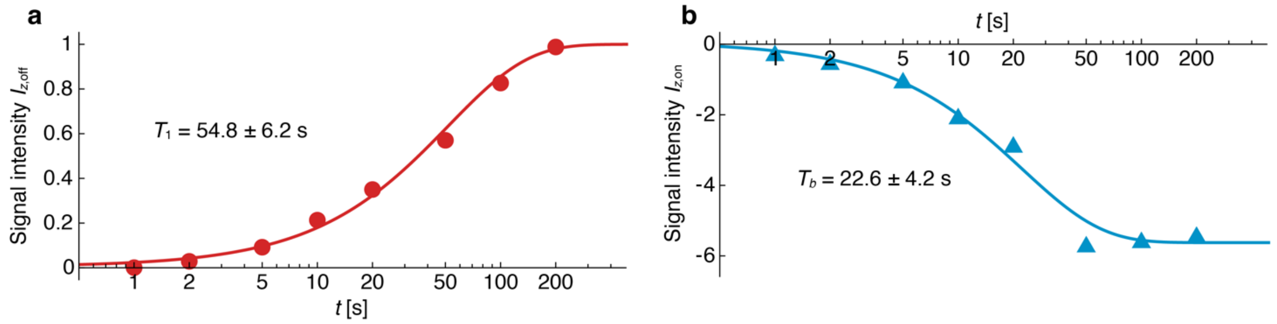

Figure S2. Saturation recovery experiments plotting the ^{1}H NMR signal intensity without light (**a**) and with CW 2.4 W/cm^2 450 nm light (**b**) as a function of re-polarization delay at 125 K for a 1 mM frozen solution of (**1**) in OTP at 0.3 T. The number of scans for each datapoint is given in Table S1. Data were fitted (solid lines) with a single exponential function having a time constant T_1 (longitudinal relaxation with no laser) or T_b (polarization buildup under CW irradiation) to yield the values given inset. All data shown here are background subtracted. Note the smaller *y* scale in (**b**) compared to that in Fig. 5b of the main manuscript.

Buildup experiments with and without 2.4 W/cm^2 laser irradiation were also performed on a 1 mM frozen solution of (**2**) in OTP at both 85 K and 125 K. Data are reported in Fig. S3, along with the fitted values for T_1 and T_b. Notably, sample degradation in the laser-on experiments at 125 K appeared to be much slower for (**2**) than for (**1**). We did not investigate this further.

For all longitudinal relaxation and polarization buildup measurements, raw NMR data were fitted with an exponential function of the form $I(t) = I_0(1 - \exp[-t / T_i]) + \delta$, with T_i equal to T_1 or T_b and δ being an offset value to account for unsuppressed probe acoustic ringing that could affect the NMR signal intensities and whose contribution has been assumed to be constant

within each series. Data reported in Figures S2 and S3 and in Fig. 5 of the main text are background subtracted (namely, raw data minus δ), and the signal enhancements in Fig. 5c have been computed as $I_{z,\text{on}}/I_{z,\text{off}}$ using the background subtracted intensities.

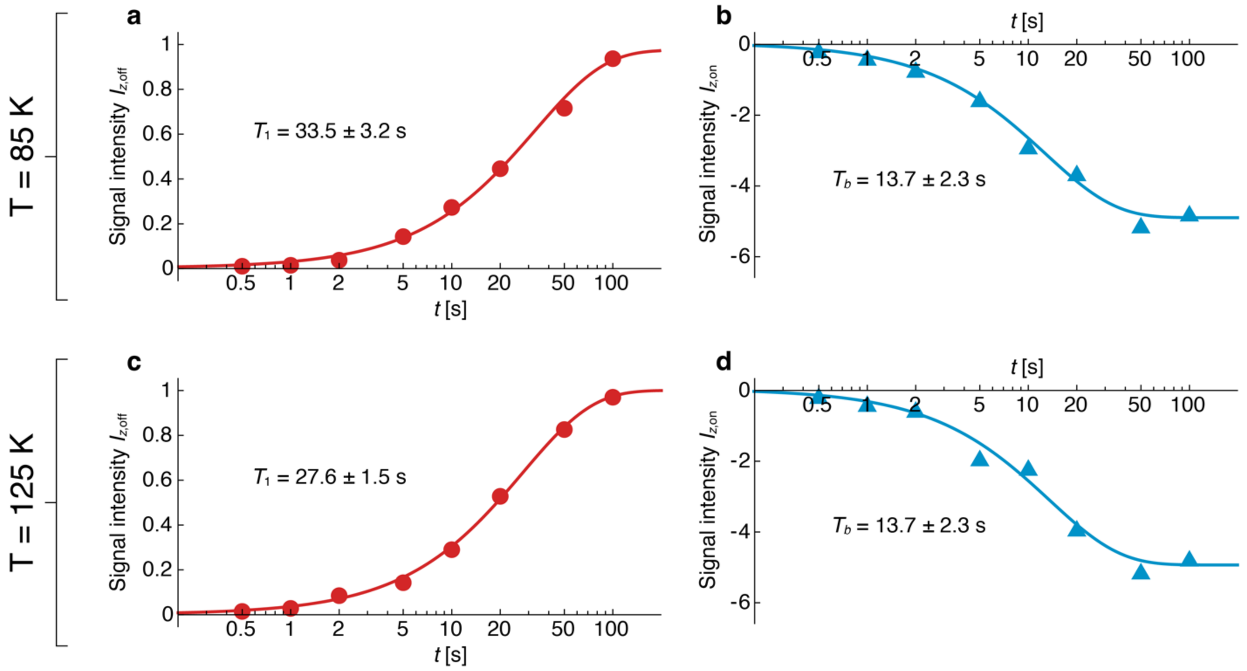

Figure S3. Saturation recovery experiments plotting the ^{1}H NMR signal intensity without light (**a**, **c**) and with CW 2.4 W/cm^2 450 nm light (**b**, **d**) as a function of re-polarization delay at 85 K (**a**, **b**) and 125 K (**c**, **d**) for a 1 mM frozen solution of (**2**) in OTP at 0.3 T. The number of scans for each datapoint is given in Table S2. Data were fitted (solid lines) with a single exponential function having a time constant T_1 (longitudinal relaxation with no laser) or T_b (polarization buildup under CW irradiation) to yield the values given inset. All data shown here are background subtracted.

Table S1. Number of scans used for each datapoint in the buildup experiments of 1 mM (1) in OTP at 85 K and 125 K and acquisition order of the laser-on series.

τ_{rec} delay	n° scans laser-off 85 K	n° scans laser-on 85 K	Laser-on acquisition order 85 K	n° scans laser-off 125 K	n° scans laser-on 125 K	Laser-on acquisition order 125 K
1 s	50,000	1,000	8	50,000	1,000	4
2 s	30,000	600	5	30,000	600	6
5 s	15,000	300	2	15,000	300	9
10 s	8,000	160	9	8,000	160	2
20 s	2,000	40	1	2,000	40	5
50 s	800	16	4	800	16	1
100 s	800	16	7	800	16	3
200 s	300	6	3	300	6	7

Table S2. Number of scans used for each datapoint in the buildup experiments of 1 mM (2) in OTP at 85 K and 125 K and acquisition order of the laser-on series.

τ_{rec} delay	n° scans laser-off 85 K	n° scans laser-on 85 K	Laser-on acquisition order 85 K	n° scans laser-off 125 K	n° scans laser-on 125 K	Laser-on acquisition order 125 K
0.5 s	96,000	4,000	8	96,000	4,000	7
1 s	28,800	1,200	4	28,800	1,200	2
2 s	7,200	300	2	7,200	300	6
5 s	2,400	100	7	2,400	100	1
10 s	1,200	50	1	1,200	50	8
20 s	720	30	5	720	30	4
50 s	480	20	3	480	20	3
100 s	480	20	6	480	20	5

3) Calculation of the spin diffusion length in OTP

The ^{1}H spin diffusion coefficient (D) in fully protonated OTP has been estimated from the measured spin diffusion coefficient of ^{19}F in CaF_2 || [001] reported in reference [88] of the main text, assuming a scaling law $D \propto c^{1/3}\, \gamma^2$ (c is the nuclei concentration in the matrix, and γ is their gyromagnetic ratio) and ignoring any potential magnetic field dependence. From this, $D_{1H,OTP} \cong 750$ nm^2/s. The spin diffusion length in OTP at 85 K in the presence of 1 mM (**1**) has been calculated as $\lambda = \sqrt{DT_1} \cong 190$ nm, as reported in the main text.

4) Steady-state photo-CIDNP experiments on (1) at 0.1 mM in OTP

Fig. S4 reports the ^{1}H NMR spectra of a frozen solution of 0.1 mM (**1**) in OTP at 85 K recorded at steady-state conditions ($\tau_{rec} = 200$ s) with and without 3.8 W/cm^2 laser irradiation ($\varepsilon = -8 \pm 1$).

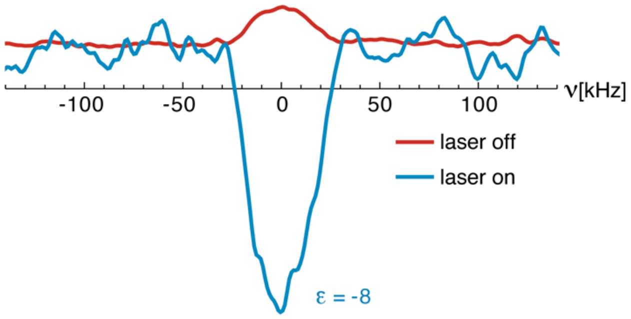

Figure S4. ^{1}H NMR spectra (12.8 MHz) of a 0.1 mM frozen solution of (**1**) in OTP at 85 K and 0.3 T without (red, 300 scans) and with (blue, 8 scans) CW 3.8 W/cm^2 laser illumination at 450 nm. The re-polarization delay between scans was 200 s, corresponding to steady-state polarization.

5) NMR experiments in other solid matrices

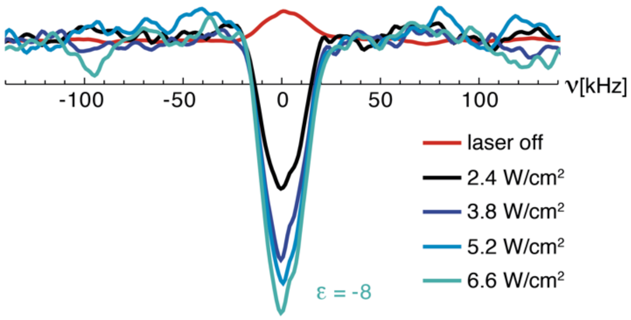

Figure S5. ^{1}H NMR spectra (12.8 MHz) of a 1 mM frozen solution of (**2**) in 20% OTP–80% OTP-d_{14} at 85 K and 0.3 T without (red, 7000 scans) and with (black and various shades of blue, 200 scans) CW illumination at 450 nm and various laser intensities. The re-polarization delay between scans was 20 s. Here the effect of light intensity was attributed to a sub-optimal alignment of the sample with the laser beam. Maximum polarization was achieved with 6.6 W/cm^2 laser intensity.

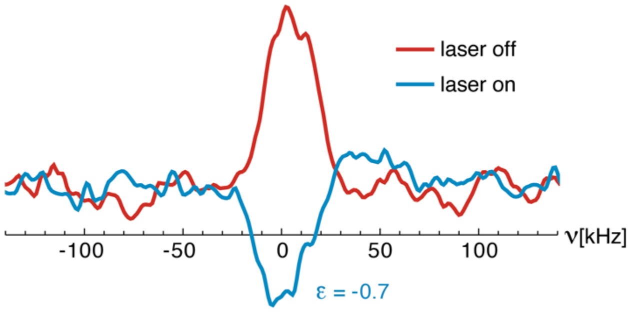

Figure S6. ^{1}H NMR spectra (12.8 MHz) of a 1 mM frozen solution of (**2**) in toluene-d_3 at 85 K and 0.3 T without (red, 200 scans) and with (blue, 200 scans) CW 5.2 W/cm^2 laser at 450 nm. The re-polarization delay between scans was 5 s.

6) Synthetic procedures

General procedures. Unless otherwise noted, all reactions were carried out under an inert atmosphere of argon. Chemicals were used as received from the suppliers. Reactions were monitored by thin-layer chromatography (TLC) analysis. Column chromatography was carried out on silica gel (230-400 mesh). The ^{1}H NMR spectra were recorded on a Bruker AVL spectrometer at 300 MHz and ^{13}C NMR spectra were recorded at 75 MHz. The chemical shifts are reported in ppm downfield relative to TMS and referenced using the residual $CHCl_3$ resonance (δ = 7.26) for ^{1}H NMR and the central $CDCl_3$ resonance (δ = 77.16) for ^{13}C NMR. ESI-HRMS were performed on a SYNAPT G2 HDMS (Waters). The NMR and MS analysis were performed at the Spectropole facilities, Marseille.

6a) Synthesis of compound <u>1</u>

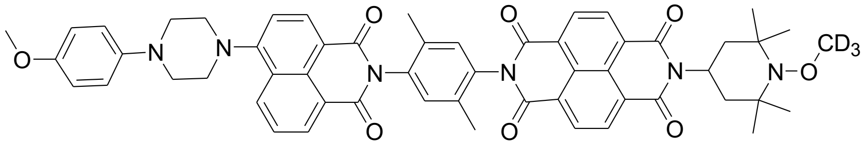

<u>1</u>

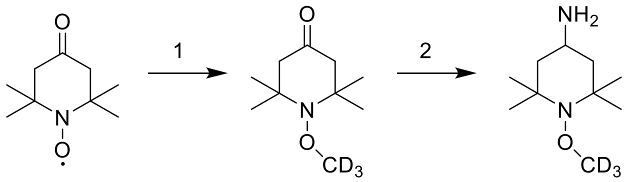

<u>A</u> **<u>B</u>** **<u>C</u>**

Scheme S1. Reagents and conditions: 1) $FeSO_4.7H_2O$, H_2O_2 (35%), DMSO-d_6, 2h, 25 °C. 2) NH_4OAC, $NaBH_3CN$, EtOH, 24h, 25 °C.

Synthesis of compound **<u>B</u>**

A 30% aqueous H_2O_2 solution (1.6 mL) was slowly added under stirring conditions to a solution of 4-oxo-2,2,6,6-tetramethylpiperidine-N-oxyl (compound **<u>A</u>**, 1.24 g, 7.29 mmol) and $FeSO_4.7H_2O$ (4.00 g, 14.38 mmol) in DMSO-d_6 (20 mL). After 2 h, 20% aqueous NaOH solution was added and the mixture was extracted twice with diethyl ether (2x 50 mL). The organic phase was dried over Na_2SO_4 and concentrated under reduced pressure. The yellow solid was purified by SiO_2 column chromatography using CH_2Cl_2/EtOH (100% to 97/3) as eluent to provide alkoxyamine **<u>B</u>** (1.05 g, 76%).

ESI-MS = 189.2 $[M+H]^+$; 211.1 $[M+Na]^+$; 399.3 $[2M+Na]^+$.

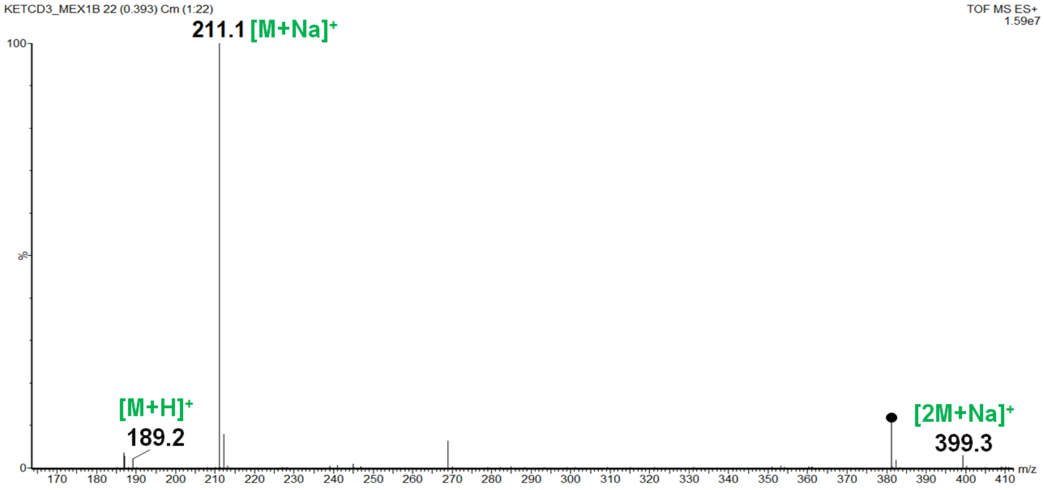

Figure S7. ESI-MS spectrum of compound **<u>B</u>**.

Synthesis of compound **C**

Compound **B** (0.35 g, 1.86 mmol) and NH_4OAc (1.45 g, 18.83 mmol) were dissolved in ethanol (10 mL) and the solution was stirred under argon atmosphere at room temperature. After 6 h, $NaBH_3CN$ (82.0 mg, 1.30 mmol) was added and the reaction was continued overnight at 25 °C. Then, a saturated aqueous solution of $NaHCO_3$ was added and the mixture was extracted twice with CH_2Cl_2, dried over Na_2SO_4 and concentrated under reduced pressure. The residue was purified by column chromatography using CH_2Cl_2/EtOH (100% to 90/10%) as eluent to provide alkoxyamine **C** (0.19 g, 54%).

^{1}H NMR ($CDCl_3$, 300 MHz) δ = 1.12 (s, 6H), 1.19 (s, 6H), 1.29 (m, 2H), 1.65 (m, 2H), 3.00 (m, 1H).

^{13}C NMR ($CDCl_3$, 75 MHz) δ = 20.73, 33.0, 42.0, 49.8, 59.8, 65.3 (septet).

ESI-MS = 190.2 $[M+H]^+$; 196.2 $[M+Li]^+$.

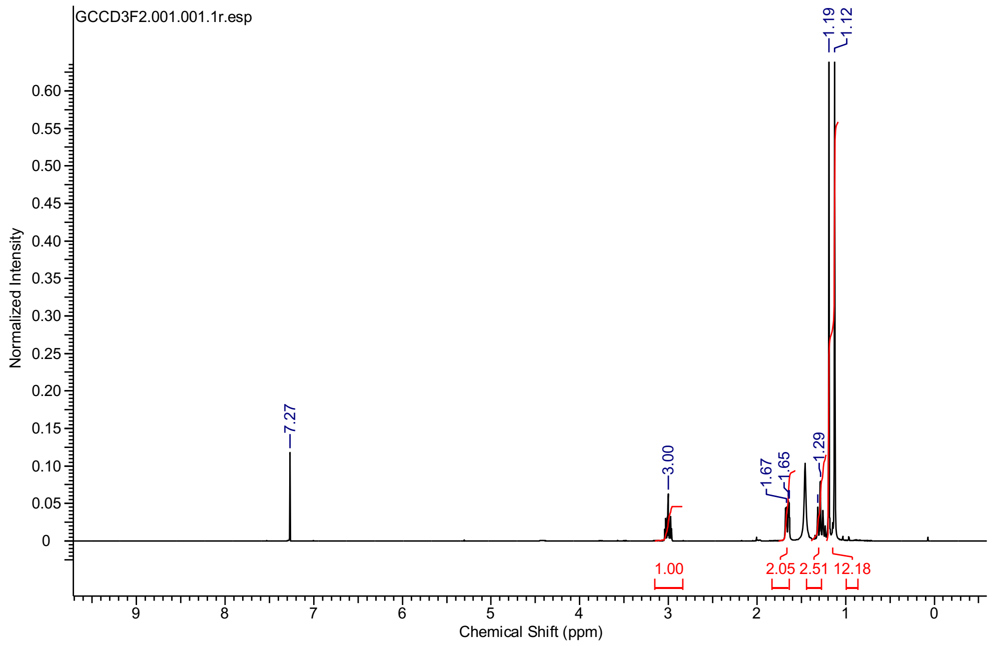

Figure S8. ^{1}H NMR spectrum of compound **C** in $CDCl_3$ at 298 K.

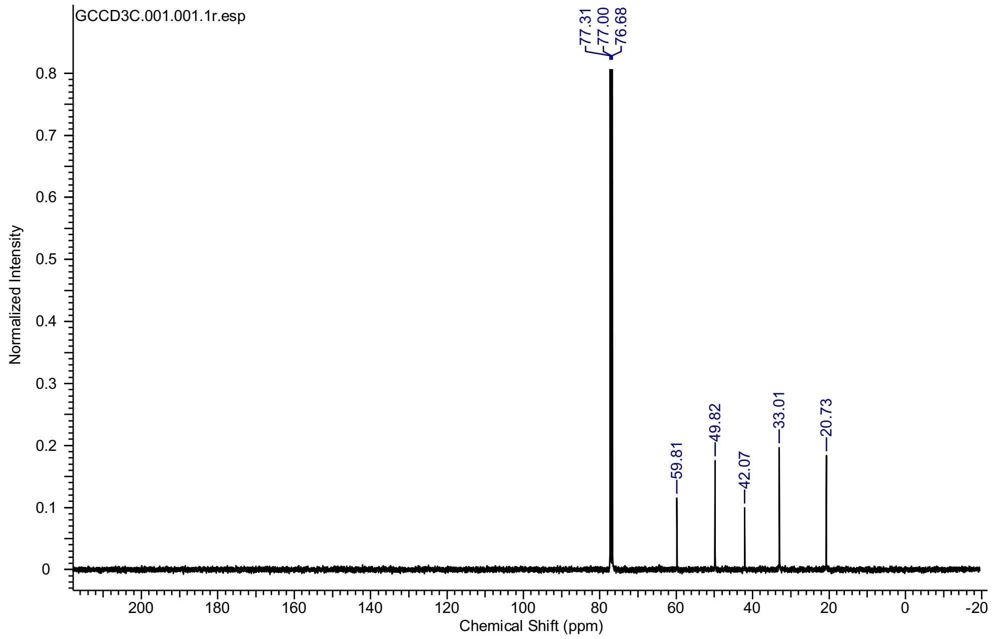

Figure S9. ^{13}C NMR spectrum of compound **C** in $CDCl_3$ at 298 K.

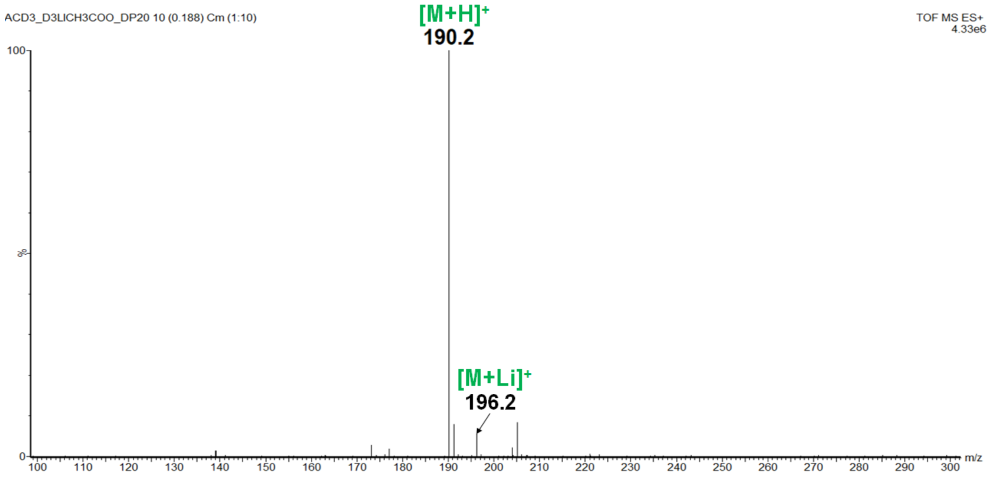

Figure S10. ESI-MS spectrum of **C**.

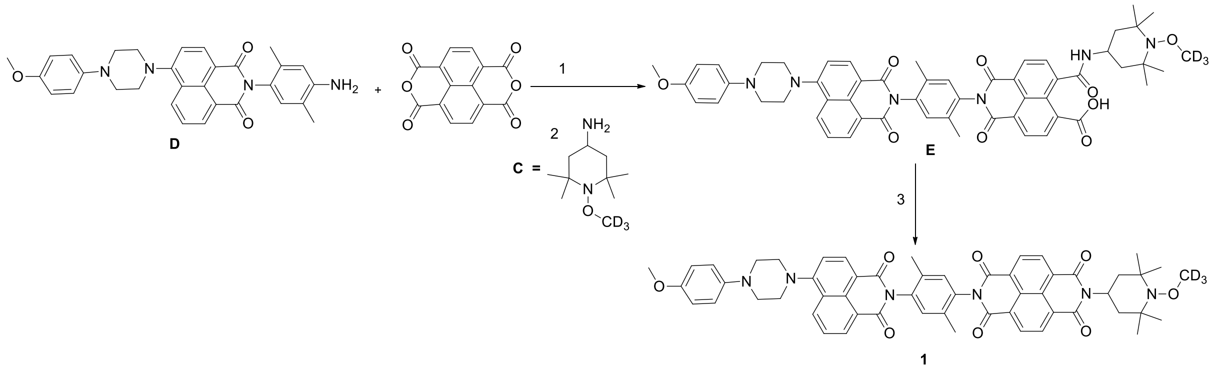

Scheme S2. Reagents and conditions: 1) Pyridine, 110 °C, 2 h. 2) Compound **C**, 110 °C, 3 h. 3) Ac_2O, $CHCl_3$, 75 °C, 4 h.

Synthesis of compound **E**

Compound **D** (50 mg, 0.10 mmol) and 1,4,5,8-naphthalenetetracarboxylic dianhydride (32 mg, 0.12 mmol) were dissolved in 3 mL of pyridine and the mixture was heated at 110 °C under an argon atmosphere for 2 h. Then, compound **C** (20 mg, 0.1 mmol) in 0.1mL pyridine was added and the reaction mixture was kept at 110 °C for 3 h. The reaction mixture was concentrated under vacuum and purified by column chromatography to give compound **E** (25 mg, 26%), $R_f = 0.35$ (10% MeOH/CH_2Cl_2).

^{1}H NMR ($CDCl_3$, 300 MHz) δ = 1.18 (s, 12H), 1.36-1.43 (m, 2H), 1.77-1.84 (m, 2H), 2.00 (s, 6H), 3.15-3.33 (m, 9H), 3.6 (s, 3H), 6.73 (d, J = 9 Hz, 2H), 6.87 (d, J = 9 Hz, 2H), 7.09 (s, 1H), 7.10 (s, 1H), 7.18-7.22 (m, 2H), 7.58-7.7 (m, 2H), 8.36-8.49 (m, 5H).

HRMS (ESI-TOF) m/z: 944.4 $[M-H]^-$; 946.4 $[M+H]^+$; 990.4 $[M-H+2Na]^+$.
$[M + Na]^+$ calculated for $C_{55}H_{51}D_3N_6O_9Na^+$ 968.4035, found 968.4033.

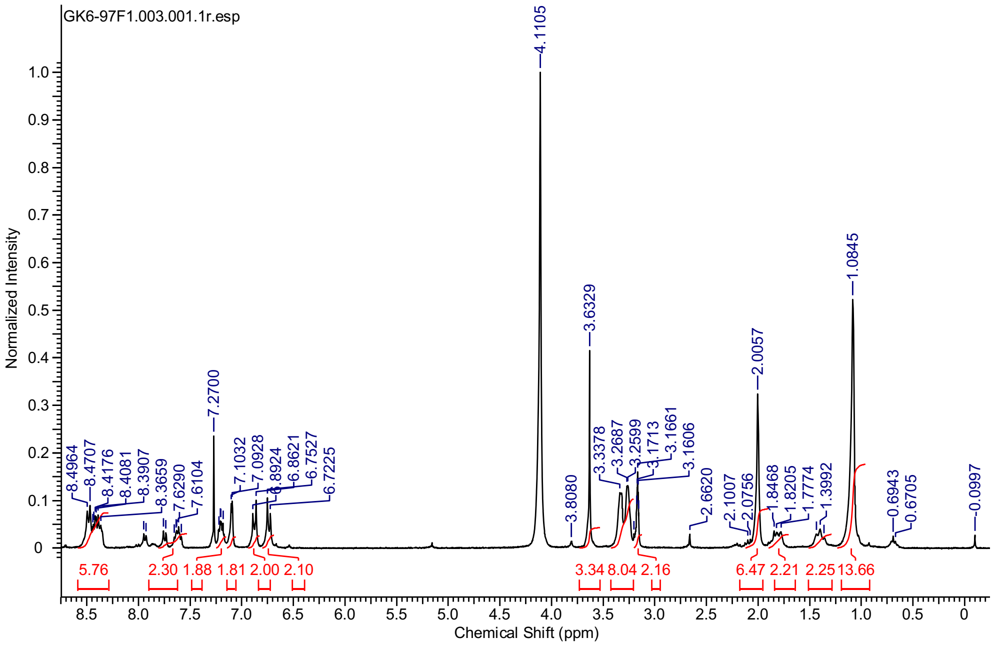

Figure S11. ^{1}H NMR Spectrum of compound **E** in $CDCl_3$ at 298 K.

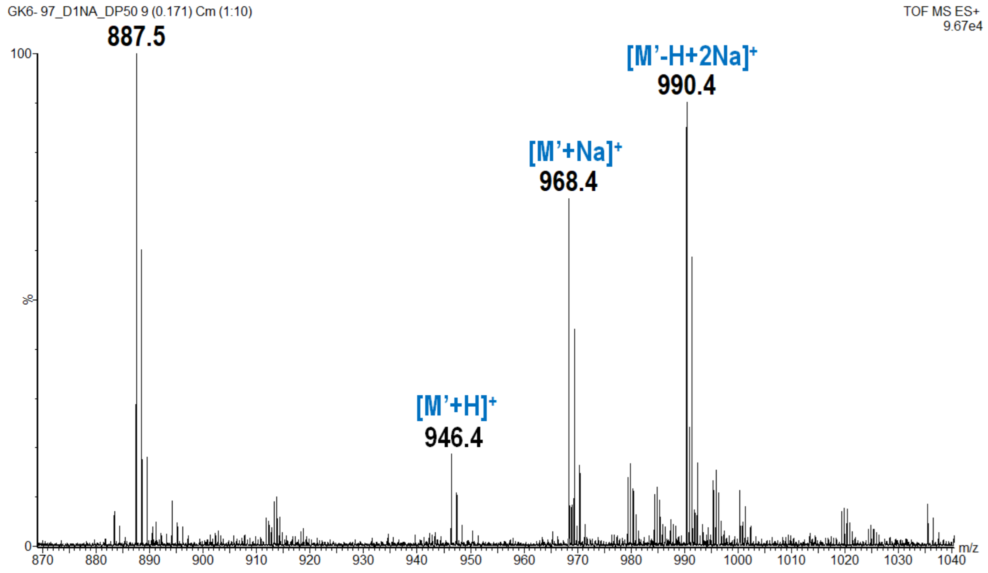

Figure S12. ESI-HRMS spectrum of **E** (positive mode).

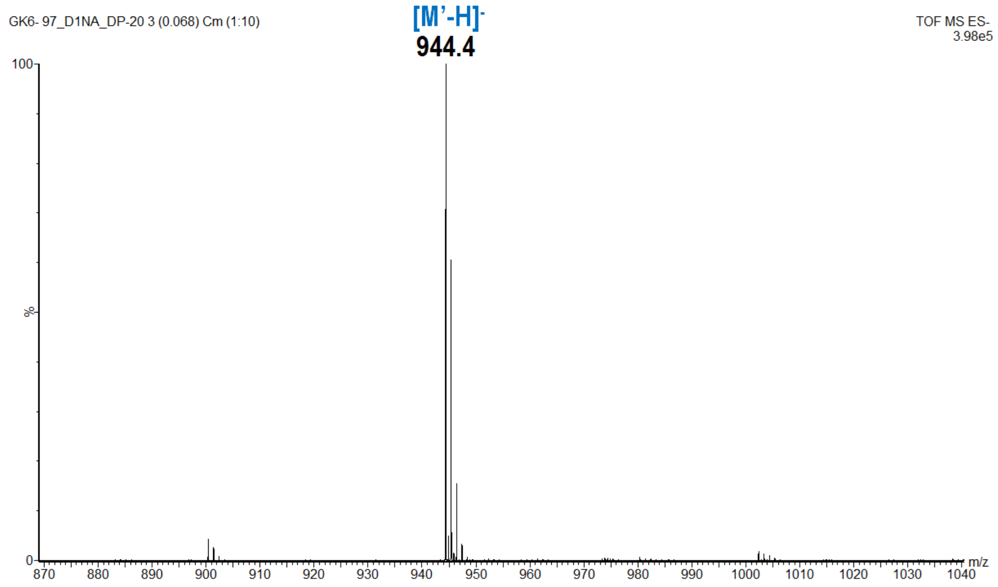

Figure S13. ESI-HRMS spectrum of **E** (negative mode).

Compound **E** (10 mg, 0.0105 mmol) in $CHCl_3$ was treated with acetic anhydride (20 µL) and heated at 75 °C for 4 h. The reaction mixture was concentrated under vacuum and purified by preparative thin layer chromatography to give **1** (4 mg, 41%), R_f = 0.7 (10% MeOH/CH_2Cl_2).

HRMS (ESI-TOF) m/z: 928.4 $[M+H]^+$; 945.4 $[M+NH_4]^+$.
$[M + Na]^+$ calculated for $C_{55}H_{49}D_3N_6O_8Na^+$ 950.3927; found 950.3939.

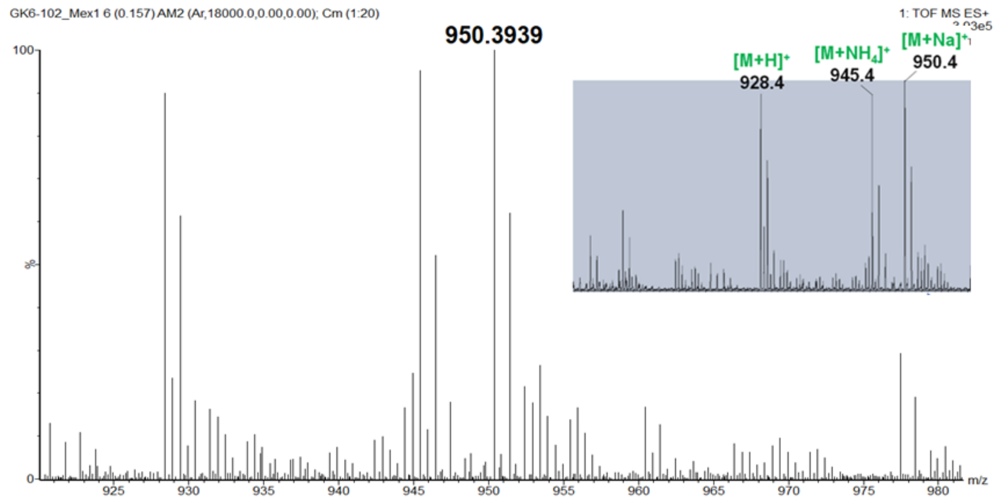

Figure S14. ESI-HRMS spectrum of compound **1**.

6b) Synthesis of compound 2

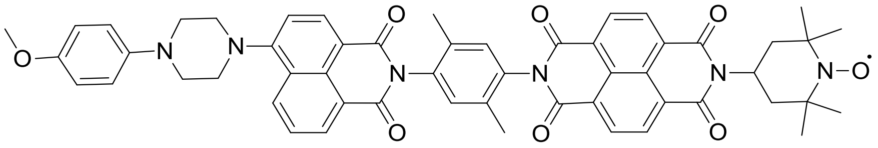

2

Compound **2** was prepared according to the published procedure in reference [76] of the main text. Compound **2** was obtained as a dark yellow solid (10 mg, 18% yield), R_f = 0.68 (10% MeOH/CH_2Cl_2).

ESI-MS: m/z: 910.5 $[M + H]^+$, 927.5 $[M + NH_4]^+$.
EPR (9 GHz, 0.4 mM in CH_2Cl_2 at 298 K) : A_N = 1.6 mT

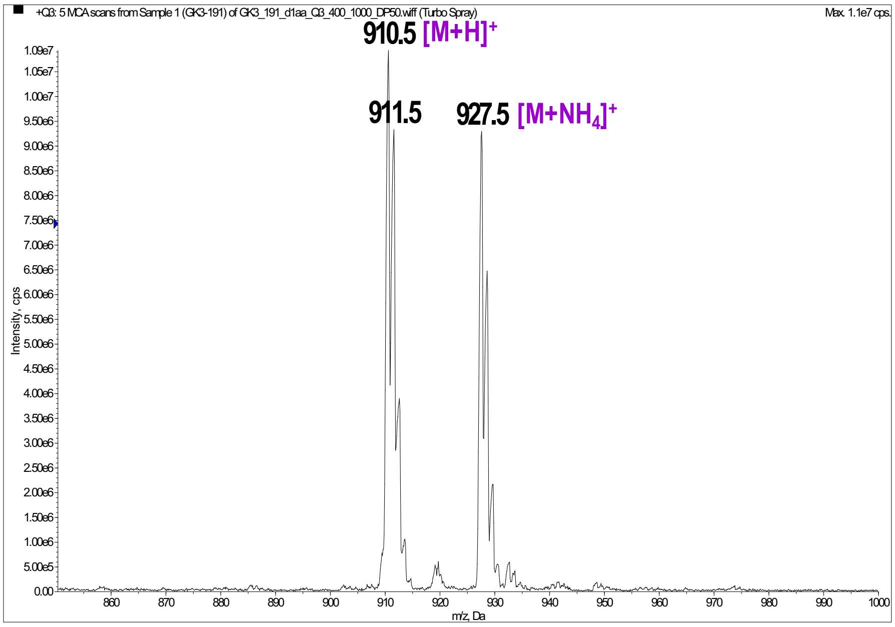

Figure S15. ESI-MS spectrum of **2**.

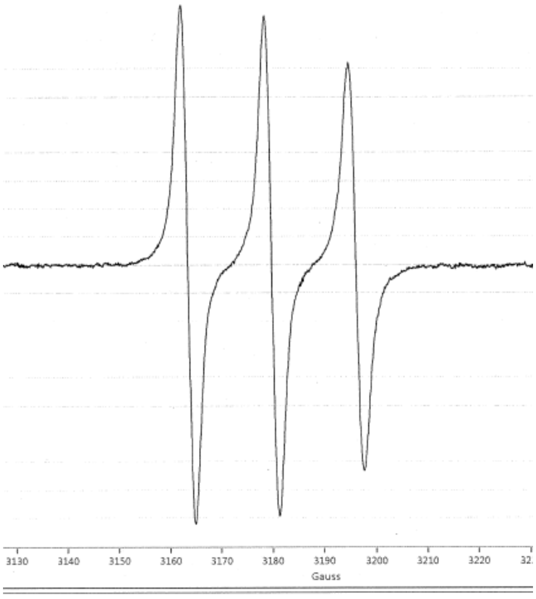

Figure S16. EPR spectrum of **2** in CH_2Cl_2 (0.4 mM) at 298 K.
